# Single-Molecule Peptide Discrimination via Flow-Through SERS and Machine Learning

**DOI:** 10.1021/acsphotonics.5c02676

**Published:** 2026-03-20

**Authors:** Kirill Khabarov, Ilaria Micol Baldi, Maria Blanco Formoso, Foroogh Khozeymeh Sarbishe, Veronica Storari, Federica Villa, Francesco Difato, Francesco Tantussi, Francesco De Angelis

**Affiliations:** † 121451 Istituto Italiano di Tecnologia, Via Morego 30, 16163 Genova, Italy; ‡ Department of Physics, University of Genova, Via Dodecaneso, 33, 16146 Genova, Italy; § Dipartimento di Elettronica, Informazione e Bioingegneria, 18981 Politecnico di Milano, Piazza Leonardo da Vinci, 32, 20133 Milano, Italy

**Keywords:** single-molecule SERS, plasmonic membrane, nanopore translocation, peptide discrimination, machine learning, SPAD camera

## Abstract

Peptides are key biomolecules in biology and medicine, yet their reliable detection and discrimination in complex mixtures remain highly challenging, particularly under clinical requirements of robustness and accuracy. Surface-enhanced Raman spectroscopy (SERS) offers molecular specificity but is hindered by spectral overlap, variability, and fluctuations that limit its applicability in practical settings. In this work, we investigate the performance of SERS flow-through strategy using plasmonic nanopores to record Raman spectra from single molecules as they translocate one by one through sub-2 nm hotspots. As a stringent test, we investigated the discrimination of vasopressin and oxytocin, two peptides differing by only two amino acids. Using electrophoretic delivery and ultrafast SERS detection with a single-photon avalanche diode camera, we captured spectra on microsecond time scales. Machine-learning analysis achieved 70.5% classification accuracy at the single-peptide level, rising to 99% discrimination when averaging 40 events. These results establish flow-through nanopore SERS as a promising route toward single-molecule peptide identification in biomedical settings.

## Graphical abstract

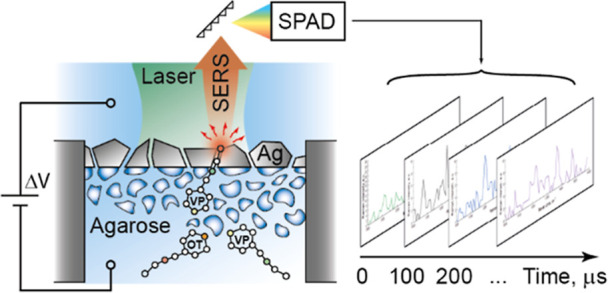

## Introduction

Peptides play a central role in biology and medicine, both as natural biomolecules and as therapeutic agents.^1^ Their detection and discrimination remain highly challenging, particularly when aiming for analytical techniques compatible with clinical settings, which demand accuracy and robustness.^2^ Conventional biochemical and spectroscopic methods often lack the speed, sensitivity, or selectivity to distinguish peptides in complex mixtures. Surface-enhanced Raman spectroscopy (SERS) is a powerful approach thanks to the molecular specificity of the Raman fingerprint, which can discriminate closely related biomolecules.^3,4^ Yet peptides present additional difficulties: their Raman spectra are relatively simple, and in clinical samples, different species mix, producing superimposed spectra that are difficult to disentangle.^5^ One could ideally build a reference spectral database, but in practice, this approach is undermined by variability and spectral fluctuations, likely caused by nonuniform adsorption on SERS substrates and random motion over the substrate surface.^6^ Such variability makes reference-based discrimination unreliable under stringent conditions and less effective. In fact, the need for spectral averaging to mitigate fluctuations smooths out spectral variations, potentially obscuring critical details that could aid in more precise molecular identification.^3^ As a result, despite decades of research and huge potential applications, SERS has not yet delivered results exploitable in clinics.^7^ In contrast, different classes of sensors, both optical and electronic, reached an outstanding limit of detection.^8,9^ For example, the group of Prof. Torsi showed single/few molecules detection via different sensing technologies.^10,11^ However, all these sensors require a label, while for many analytes, especially peptides and proteins, labels are not available or are expensive.

An alternative SERS strategy consists of using plasmonic nanopores, where molecules flow one by one through the hotspot by means of electrophoresis. The approach also enables sequential spectra from individual molecules and avoids ensemble mixing.^12–15^ Because molecules are not adsorbed on the surface, they are less deformed by surface interaction and are excited by more homogeneous fields, yielding more reproducible spectra. Using this approach, different groups independently showed that plasmonic nanopores can discriminate both single nucleotides^4^ and amino acids (AA)^13,16^ during translocation. The plasmonic hot spot, generated inside the pore, functions as an optical probe reaching a resolution of a few AA or nucleotides and, in specific cases, enables the detection of even single-point mutations.^13^

These methods are inspired by solid-state nanopore DNA sequencing.^17,18^ It is best suited for linear or linearizable molecules such as DNA, RNA, and proteins, which are long enough to pass longitudinally and be read out step by step. Shorter biomolecules like peptides, however, pose further challenges: with sizes of 2–3 nm, comparable to the pore itself, they pass in random orientations, so their spectra cannot be segmented but are recorded in full, similar to conventional SERS. Nevertheless, the nanopore approach retains key advantages: it measures one molecule at a time, avoids spectral overlap, and yields more stable spectra. Combined with modern machine-learning (ML) tools,^19^ this enables powerful single-molecule comparative analyses that go beyond the limits of conventional SERS.

In this work, we analyze the ability of plasmonic nanopores and the flow-through strategy to discriminate minor differences in molecules whose size is smaller than the nanopore in every direction. As test molecules, we investigated oxytocin and vasopressin, which consist of 9 AA and differ only by two residues in the third and eighth positions. We delivered the molecules into the plasmonic pores via electrophoresis and tracked their translocation spectra by collecting data with 100 μs time resolution. For capturing such a fast SERS dynamic,^20^ we took advantage of the latest generation single-photon avalanche diode (SPAD) camera. We delivered oxytocin and vasopressin separately to build a database of single-molecule spectra suitable for ML training. Using these data sets, we applied principal component analysis (PCA) together with random forest classification and obtained a single-protein discrimination accuracy of 70.5%. Notably, the accuracy for molecular discrimination reaches 99% by collecting 40 events, which can be achieved within 5 min of data collection at 1 nM concentration. Next, we prepared molecular mixtures at 1:1 and 1:9 concentration ratios and collected the spectra under the same experimental conditions. When applying the same classification model to the spectra collected in the mixture, the model correctly retrieves the two molecular classes according to their relative concentration. These results, although still at a proof-of-concept level, demonstrate that a flow-through approach can overcome typical limitations of SERS such as spectral fluctuation. Also, the strategy provides most of the features necessary to achieve spatial resolution and sensitivity comparable to the size of a single functional group within larger molecules. This opens the way to reliable identification of single-point mutations in DNA or post-translational modifications in proteins, which remain unresolved challenges in current diagnostic and therapeutic approaches.

## Results and Discussion

Oxytocin (OT) and vasopressin (VP) are peptide hormones that play a critical role in regulating physiological and social behaviors such as stress responses, bonding, and cardiovascular regulation.^21–23^ Moreover, VP also serves as a valuable biomarker present in plasma for the diagnosis of central diabetes insipidus.^24^ To analyze their subtle differences, we studied SERS spectra for the single peptides in a flow-through geometry using a permeable plasmonic membrane consisting of a nanostructured silver layer sputtered onto an agarose medium capable of guiding molecules through. The porous nature of agarose leads to the formation of a polycrystalline silver film with intrinsic nanopores (slits and holes) between individual crystals that form highly localized hot spots (Figure 1a). Fabrication details are reported in Supporting Information Note 1, while a detailed characterization of the pore size and morphology is provided in our previous work.^4^ When an electrophoretic field is applied, molecules migrate first through the gel, much like in conventional electrophoresis, before moving through the silver film, where their interaction with the plasmonic surface is enhanced.

**Figure 1:**
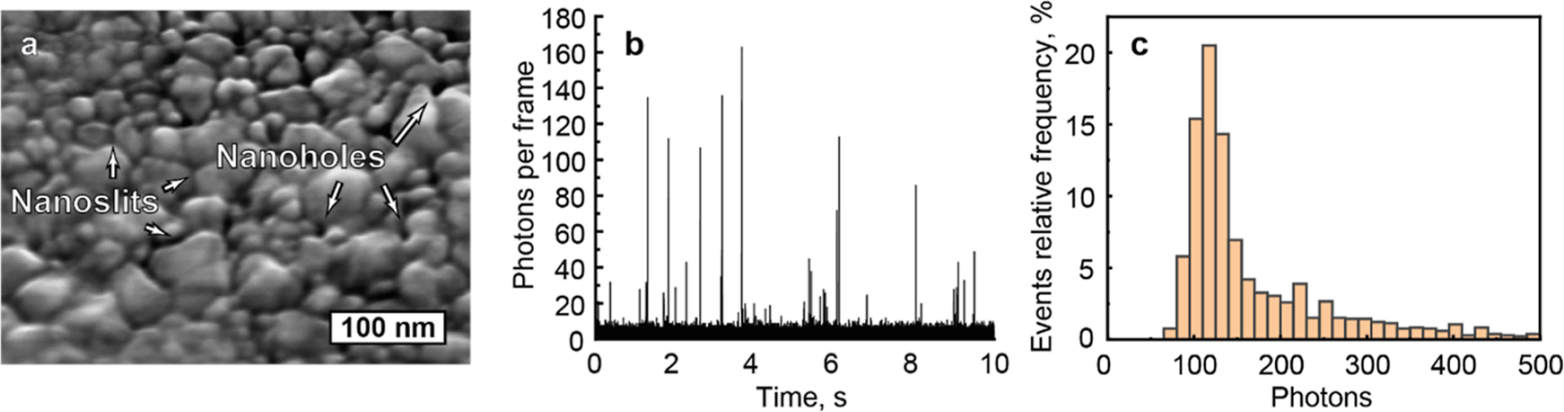
System description and performance: (a) SEM image showing nanopores formed between silver crystal grains on top of the agarose gel. (b) Time trace of the integrated number of photons per frame spectrum. (c) Distribution of photons emitted associated with detected translocation events.

A temporal resolution on the microsecond time scale is necessary to follow the molecules’ translocation dynamics through the nanopores (Figure 1b). With this aim, we integrated a 64 × 32 pixels SPAD camera (Micro Photon Devices—MPD, HERMES Series) into a Raman microscope (InVia, Renishaw) with customized software. Compared to conventional CCD or CMOS cameras, SPAD operates in a single-photon detection regime with negligible readout noise, enabling the time-resolved detection of photons from translocation events.

Based on the previous system characterization,^4^ the acquisition time window was set to 100 μs to ensure single-molecule detection conditions. To probe the spectral response of the plasmonic film, we employed a 532 nm laser and, optimizing the system for SERS detection, set it at a power of 0.1 mW, corresponding to a power density of approximately 10 kW/cm^2^ at the focus. This value represents a compromise between the maximum number of photons excited while keeping the membrane and translocating molecules undamaged. The Raman signal was studied within a maximum allowed wavenumber range 1000–1600 cm^–1^, where the molecules show the most intense signal.^25^ The setup and detection scheme are described in greater detail in ref 4.

VP and OT were selected as model molecules due to their structural complexity and high similarity. Both peptides consist of 9 AAs, differing only at the third (isoleucine vs phenylalanine) and eighth (leucine vs arginine) positions, as shown in Figure 2a caption. Although a single AA has an approximate length of 0.4 nm, these molecules adopt a compact cyclic structure, making their effective size significantly smaller than the sum of individual residues. The molecular dimensions of these peptides are 2–3 nm in length and 1–2 nm in width, and are comparable to the average size of the used nanopores. Therefore, these peptides may translocate through the pores in multiple conformations, potentially influencing their interaction with the plasmonic surface.

**Figure 2:**
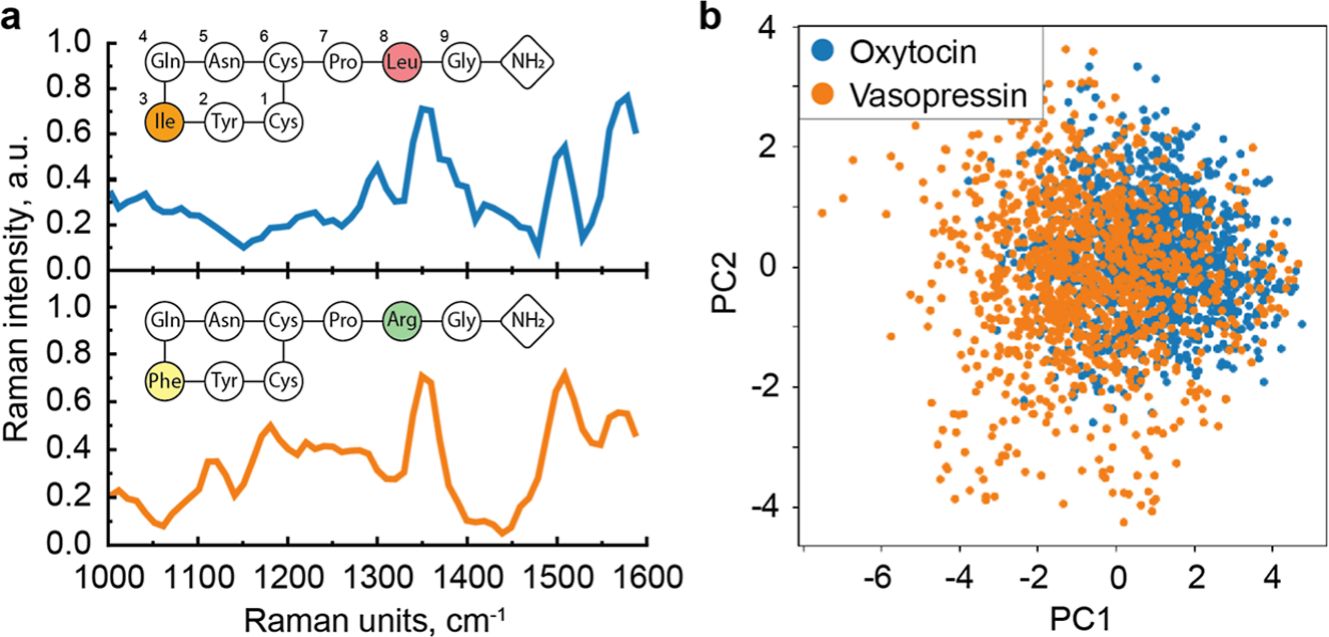
Molecules’ characterization. (a) Averaged SERS spectra of OT (up) and VP (down) with their AA structure. (b) PCA for separate translocation experiments with OT and VP for single-molecule spectra along PC1 and PC2.

For the experiments, the peptides were dissolved at a concentration of 1 nM in a 150 mM NaCl buffer solution (purity >99%), dispersed in deionized water (>18.2 MΩ·cm, Milli-Q system), and additionally filtered through a 0.22 μm filtering membrane. Under our conditions, both peptides carry a positive charge, and therefore both migrate from the negative to the positive chamber under an applied bias of 100 mV. VP is slightly more positively charged than OT due to the presence of arginine at position 8, replacing the neutral leucine in OT. These conditions are close to physiological and are commonly used in related studies.^12,13,26^

In the first experiment, peptides were studied separately to characterize their translocation through the permeable plasmonic film, analogous to electrical sequencing with a solid-state nanopore. However, instead of being detected by ionic currents, the molecules were identified and discriminated based on their unique Raman fingerprints. A key aspect of this flow-through approach is that the molecules remain free to move in a liquid environment, rather than being adsorbed onto the metal surface. As was demonstrated in our previous work,^4^ at a concentration of 1 nM, the observed translocation events are consistent with single-molecule passage through the plasmonic pores. This ensures that the recorded signals originate from freely translocating single-molecule species rather than surface-affected vibrations.

Collecting hundreds of translocation events, we calculated the total number of photons detected for each single translocation, and we found that the investigated peptides scatter approximately 120 Raman photons during the translocation time (Figure 1c). This roughly corresponds to 13 photons per AA, and it is in good agreement with the previously reported data.^4^ In Figure 2a, we present the average SERS spectra of the two peptides. These spectra were processed using the custom-created Python code through the procedure described next.

First, the raw spectral data were cleaned by removing damaged detector channels (pixels) previously identified as faulty. Each temporal frame of the spectral data set was then smoothed using a Wiener filter with a predefined window size in order to reduce random noise, preserving persistent spectral features. Then, to identify molecular translocation events, a time trace was constructed by summing the photon counts across all spectral channels for each time frame (Figure 1b). To eliminate background intensity fluctuations, the signal was divided into nonoverlapping segments of fixed length. A local median baseline was computed and subtracted from each segment to compensate for slow background fluctuations. A threshold was then applied to the time trace graph to identify the intervals corresponding to individual translocation events. For each identified event, the photon counts were summed over the event duration for each detector channel (pixel) to obtain a single-molecule SERS spectrum. The cumulative spectra (Figure 2a), representing the average for many single-molecule SERS spectra, therefore reflect exclusively the molecular signals, assuming that impurities in the solution are minimal.

As expected, the spectra exhibit high similarity, although they remain distinct and distinguishable when analyzed independently, i.e., not mixed within the same sample. To further illustrate this point, we performed PCA analysis,^27^ with results shown in Figure 2b. The data clusters associated with the two peptides show substantial overlap. However, their centroids remain separable, reflecting small but consistent differences in the average spectra. Figure S2 and Table S1 show the centroid positions and data set variances, further supporting this observation. This indicates that, at the single-protein level, i.e., for individual data points in the PCA space, spectral fluctuations remain significant. Ultimately, this prevents reliable molecular discrimination when the analytes are present in the same sample.

To further explore the variability of individual events and determine whether the studied peptides can be discriminated at the single-molecule level, we applied a Random Forest classification algorithm. The analysis followed the pipeline: spectra—preprocessing—PCA—Random Forest classification. While PCA reveals the main variance structure of the data set, Random Forest enables supervised discrimination by exploiting subtle spectral features. Before classification, the spectra were preprocessed using a PowerTransformer normalization, followed by dimensionality reduction using PCA. The first 10 principal components (PC), used as input features for the classifier, account for 27% of the variance in the transformed data set. The data set consisted of approximately 400 spectra for each peptide (OT and VP). A 70/30 train-test split was used for performance evaluation, corresponding to 280 spectra used for training and 120 used for testing. Model performance was computed over 10 iterations with a randomly selected test set in each run. Under these conditions, we achieved a consistent average accuracy of 70.5% in distinguishing OT and VP single-protein spectra acquired from separate translocation experiments. The corresponding confusion matrix and classification report with key classification metrics such as precision, F1 score, and recall are shown in Figure S3. More in detail, these parameters can be defined through the TP (True Positives), TN (True Negatives), FP (False Positives), and FN (False Negatives) values, which stand for correctly predicted positive instances, correctly predicted negative instances, incorrectly predicted positive instances, and incorrectly predicted negative instances as

1 $$accuracy=\frac{TP+TN}{TP+TN+FP+FN}$$

2 $$precision=\frac{TP}{TP+FP}$$

3 $$recall=\frac{TP}{TP+FN}$$

4 $$F1=2\times \frac{precision\times recall}{precision+recall}$$

Accumulating and averaging the single-molecule spectra, the classification becomes more reliable due to the increase in the signal-to-noise ratio. Just by collecting 40 events, which can be achieved within 5 min of data acquisition at 1 nM concentration, the accuracy for molecular discrimination reaches 99%.^4^ The classification accuracy achieved in separate experiments suggests that our method provides high discrimination power for single-molecule detection and identification. A key open question, however, is whether molecular discrimination remains effective when analytes are part of a complex mixture. To address this, we conducted an additional translocation experiment using a mixture of OT and VP dissolved at equal concentrations of 1 nM. For the current analysis, we utilized a total of three data sets: two arrays from the previous experiments with individual molecular translocations of OT and VP, and a third array containing the translocation data of the mixture. As in the previous case, PCA produced two clusters with distinct centers corresponding to the individual experiments. The spectra from the mixed sample formed a third group that was superimposed by the algorithm on top of the two existing clusters (Figure S4), making it difficult to assign the events to the correct molecular class.

As before, we applied the Random Forest algorithm for the 3 data sets following the procedure described above, which gives a better characterization than PCA only. In that approach, the classification method was distributing data from the mixed class into the classes corresponding to single-protein translocation data sets, as shown schematically in Figure 3a. We noticed that during the classification of the data into classes, the FP values (wrong predictions) for the mixture were classified into OT and VP with an equal contribution (Figure 3b). The ratio between these values corresponds to the initial loading ratio of the sample concentrations. To prove that, we performed the following calculations. Since the algorithm takes a subset of the data for training in every classification run, the ratio between FP values distributed from a mixed class to separate classes could vary. But by repeating this procedure multiple times, the maximum repeating value of those ratios will correspond to the true ratio between concentrations. This may be easily shown with a histogram containing fluctuations, described by a hypergeometric distribution (Figure 4a).

**Figure 3:**
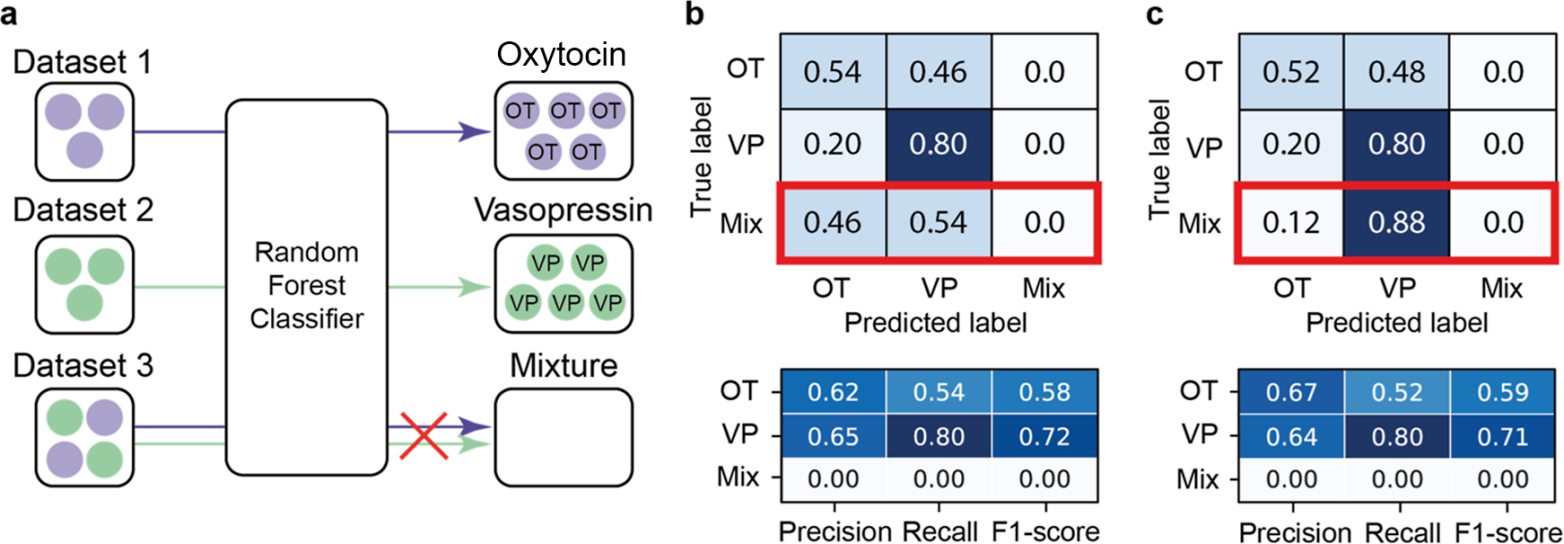
Random Forest classification of Raman spectra for OT, VP, and their mixture. (a) Schematic illustration of the approach. Confusion matrices showing the redistribution of mixture spectra into OT and VP classes in ratios of distributed molecules, along with classification metrics per class for (b) 1:1 and (c) 1:9 concentration ratios of OT and VP.

**Figure 4:**
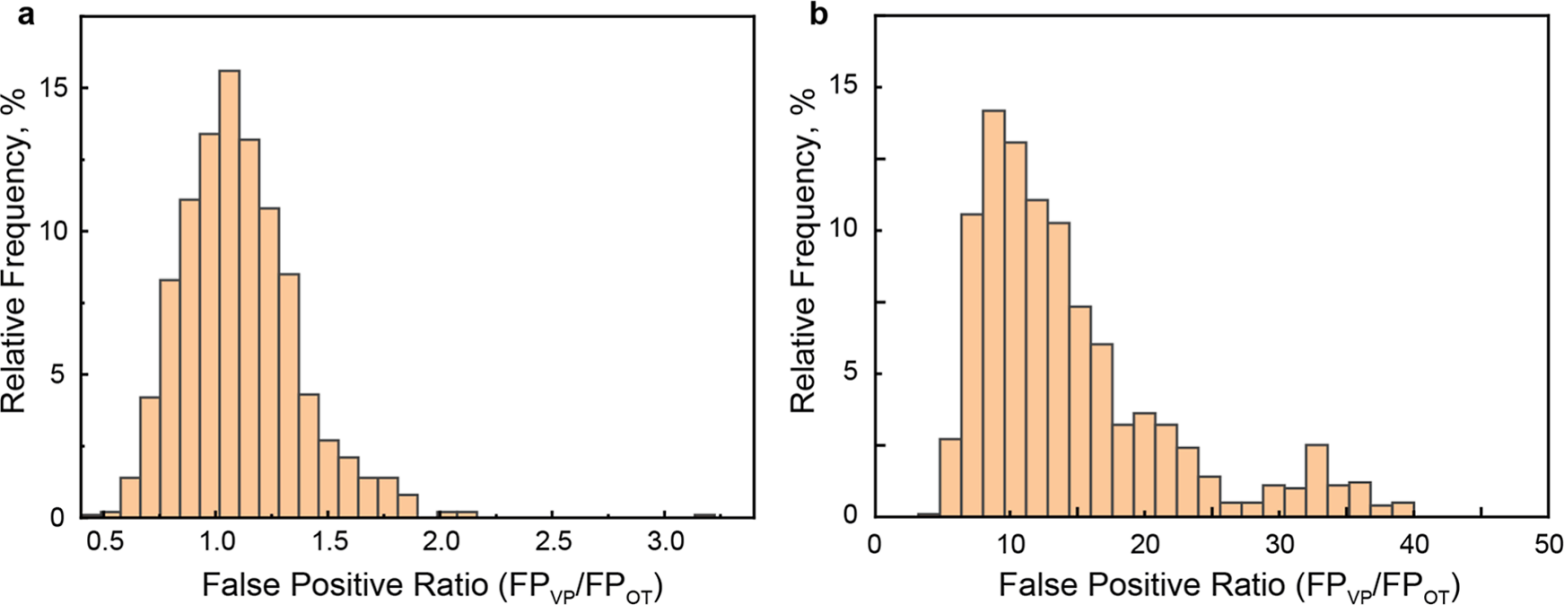
Histogram of VP/OT ratios obtained from FP assignment of mixture spectra across random seeds for the experiment with (a) 1:1 and (b) 1:9 concentration ratios of OT and VP. The histogram maximum corresponds to the true molecular composition, consistent with a hypergeometric distribution.

To support this idea, and to further validate the overall approach, we set another experiment with different concentration values for OT and VP as 1:9 (Figure 3c). And indeed, repeating the whole procedure, the final ratio turned out to be 1:8.8 (Figure 4b). The observed difference may stem from minor inaccuracies in sample preparation or from variability in translocation dynamics associated with differences in molecular charge.

The ability to discriminate between closely related peptides such as OT and VP in both separate and mixed experiments highlights the potential of our approach for high-accuracy molecular identification in complex biological environments. The method described enables the quantification of unknown concentrations in molecular mixtures, assuming the spectra of the components are known. By adding a test molecule at a known concentration to the solution and analyzing the classification outcomes, the relative abundances of each molecular species can be inferred with high accuracy. The results provided demonstrate the importance of single-molecule SERS with the proper temporal and spectral resolution for laying the potential for next-generation analytical techniques.

## Conclusions

In this study, we demonstrated the applicability of our method based on SERS for the detection and classification of single peptides with high similarity, translocating through a permeable plasmonic membrane under electrophoretic driving force. The approach uses the advantages of a nanostructured silver film on agarose to measure signals in a flow-through geometry, exploiting the benefits of gel electrophoresis, allowing label-free identification of freely moving molecules in solutions. By employing an ultrafast SPAD camera, we captured SERS spectra of individual translocation events with 100 μs resolution. Using ML tools, we achieved a classification accuracy of 70.5% for distinguishing OT and VP on a single-molecule level, despite their structural similarity. By averaging 40 events, the accuracy exceeds 99%. Importantly, the method remains effective for the mixtures of molecules and even enables estimation of concentration ratios through repeated classification runs. These results strengthen the idea that flow-through SERS strategies are more effective for single-molecule analyses in complex media and can potentially overcome the limitations that prevented SERS from reaching practical applications, including spectral fluctuations and substrate variability, which are among the most practical limitations of SERS.

## Supplementary Material



## Notes

Note 1: Preparation of the permeable plasmonic substrate. Figure S1: SEM picture of a permeable plasmonic structure. Figure S2: PCA of single-translocation spectra for the OT and VP data sets. Figure S3: Random Forest classification of Raman spectra for OT and VP. Figure S4: PCA of single-translocation spectra for the OT, VP, and their mixture data sets. Table S1: PCA parameters of single-translocation spectra for the OT and VP data sets (PDF)
