# Supplementary material for: Single-Molecule Peptide Discrimination via Flow-Through SERS and Machine Learning
Source: ACS Photonics. 2026 Mar 20;13(7):1840–5. doi: 10.1021/acsphotonics.5c02676 (PMC13047724; doi:10.1021/acsphotonics.5c02676)
Supplement: Supplementary file 1 [file ph5c02676_si_001.pdf]

Supporting Information

Single-Molecule Peptide Discrimination Via Flow-Through SERS and Machine Learning

Kirill Khabarov[1], Ilaria Micol Baldi[1,2], Maria Blanco Formoso[1], Foroogh Khozeymeh Sarbishe[1], Veronica Storari[3], Federica Villa[3], Francesco Difato[1], Francesco Tantussi[1], Francesco De Angelis[1*]

[1] Istituto Italiano di Tecnologia, Via Morego 30, 16163, Genova, Italy

[2] Department of Physics, University of Genova, Via Dodecaneso, 33, 16146 Genova, Italy

[3] Dipartimento di Elettronica, Informazione e Bioingegneria, Politecnico di Milano, Piazza Leonardo da Vinci, 32, 20133 Milano

* Corresponding author: francesco.deangelis@iit.it

4 pages, 1 note, 4 figures, 1 table

Supplementary Note 1. Preparation of the Permeable Plasmonic Substrate

Commercial Silicon nitride membranes measuring 500 × 500 μm² and 500 nm thick were milled by Focused Ion Beam (FIB) to create a well of 500 nm in diameter. A solution of agarose gel (1%, 10 μL) was then spin-coated on the membrane and allowed to solidify at room temperature for 5 min, followed by complete drying on a preheated hotplate at 60 °C for 5 min. Subsequently, 2 μL of hexamethyldisilazane (HMDS) was applied to the upper side of the membrane to remove residual moisture and improve the following metal adhesion to the agarose. After an additional oxygen plasma cleaning step (100 W, 5 min), 5 nm titanium (Ti) and 12 nm silver (Ag) were consecutively sputtered onto the membrane's upper surface. An example of the fabricated nanopores is shown in Figure S1: both the well on the membrane (500 nm in diameter) and the nanopores on silver are shown.

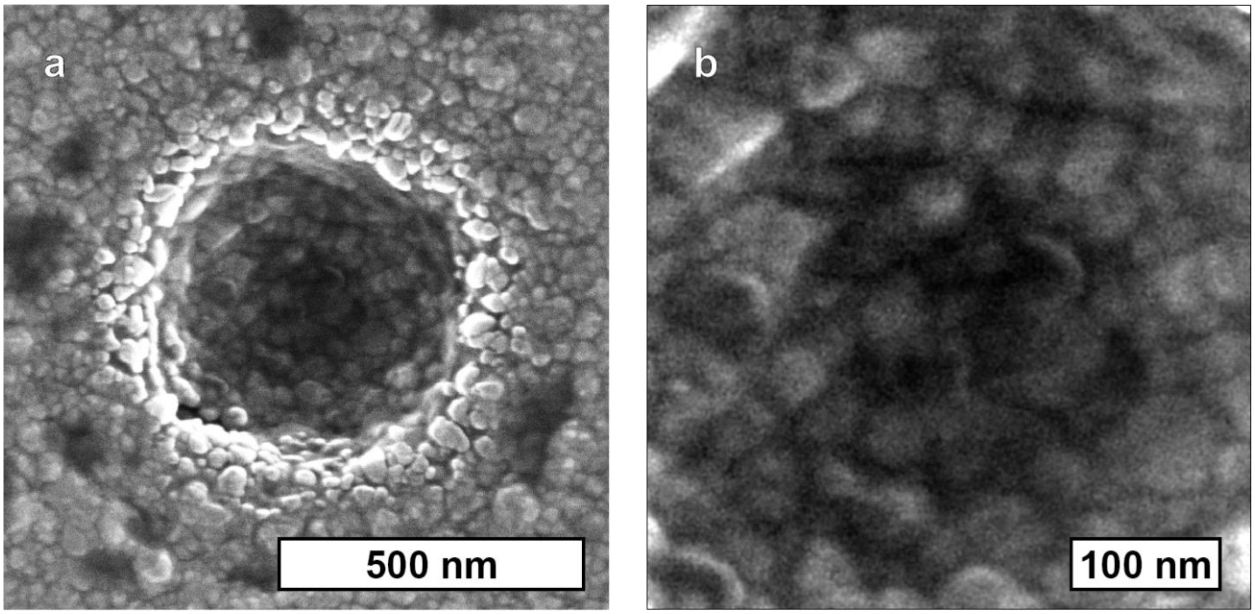

Figure S1. SEM images of a permeable plasmonic structure (top view): (a) overview of a 500 nm well; (b) magnified view of the inner structure of the well, showing silver crystals on top of the agarose gel and forming a permeable plasmonic contact.

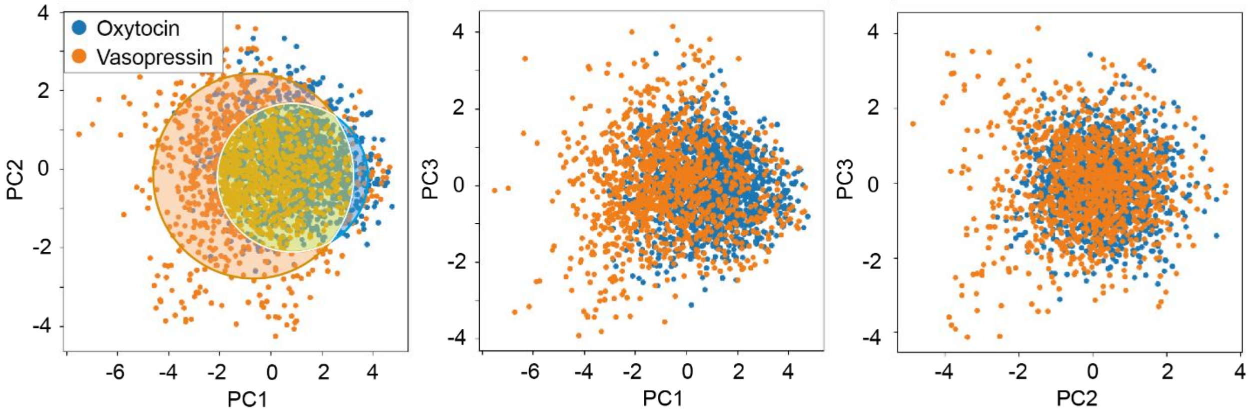

Figure S2. PCA of single-translocation spectra for the OT and VP datasets. The blue (OT) and orange (VP) circles represent the data variance, while the yellow area indicates the overlap between the two distributions.

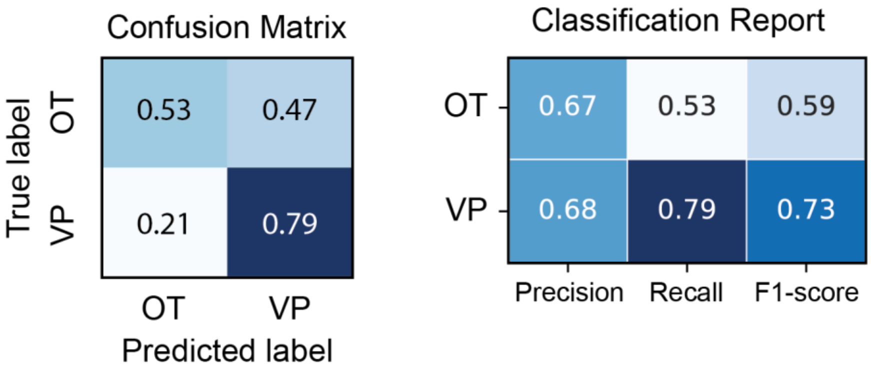

Figure S3. Random Forest classification of Raman spectra for OT and VP: confusion matrix showing the distribution ratios, and classification metrics.

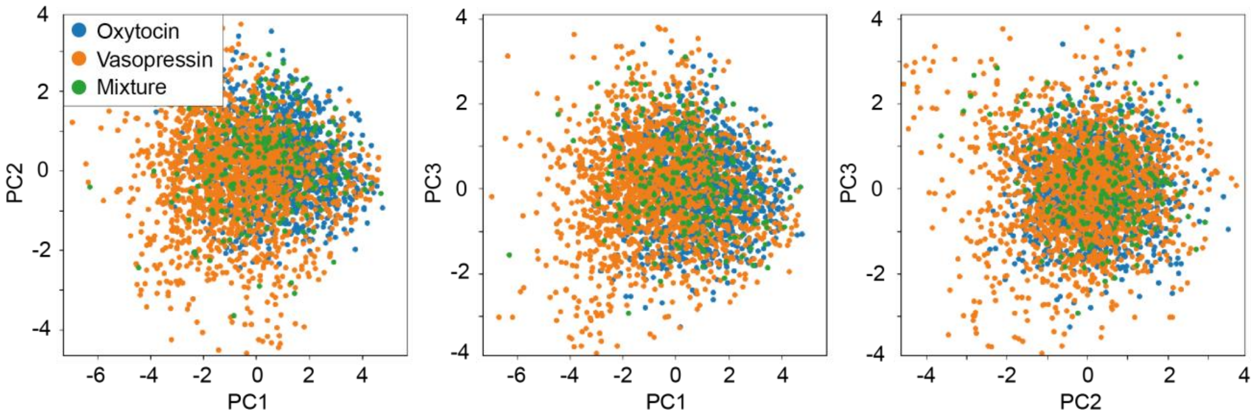

Figure S4. PCA of single-translocation spectra for the OT, VP, and their mixture datasets.

Table S1. The centroid and the variance for PC1, PC2, and PC3 for PCA of single-translocation spectra for the OT and VP datasets.

	PC1	PC2	PC3
Centroid Oxytocin	0.85	0.18	-0.04
Centroid Vasopressin	-0.67	-0.14	0.03
Variance Oxytocin	2.12	0.91	0.93
Variance Vasopressin	3.77	1.74	1.60
